# Defects in division plane positioning in the root meristematic zone affect cell organization in the differentiation zone

**DOI:** 10.1242/jcs.260127

**Published:** 2022-09-29

**Authors:** Alison M. Mills, Carolyn G. Rasmussen

**Affiliations:** ^1^ Graduate Group in Biochemistry and Molecular Biology; ^2^ Department of Botany and Plant Sciences, Center for Plant Cell Biology, Institute of Integrative Genome Biology, University of California, Riverside, CA 92521, USA

**Keywords:** Mitosis, Phragmoplast, Root twisting, Cell file rotation, Division plane orientation, *Arabidopsis*

## Abstract

Cell-division-plane orientation is critical for plant and animal development and growth. TANGLED1 (TAN1) and AUXIN-INDUCED IN ROOT CULTURES 9 (AIR9) are division-site-localized microtubule-binding proteins required for division-plane positioning. The single mutants *tan1* and *air9* of *Arabidopsis thaliana* have minor or no noticeable phenotypes, but the *tan1 air9* double mutant has synthetic phenotypes including stunted growth, misoriented divisions and aberrant cell-file rotation in the root differentiation zone. These data suggest that TAN1 plays a role in non-dividing cells. To determine whether TAN1 is required in elongating and differentiating cells in the *tan1 air9* double mutant, we limited its expression to actively dividing cells using the G2/M-specific promoter of the syntaxin *KNOLLE* (*pKN:TAN1–YFP*). Unexpectedly, in addition to rescuing division-plane defects, expression of *pKN:TAN1–YFP* rescued root growth and cell file rotation defects in the root-differentiation zone in *tan1 air9* double mutants. This suggests that defects that occur in the meristematic zone later affect the organization of elongating and differentiating cells.

## Teaser

**Summary:** Expression of *TAN1* in the root meristematic zone rescues cell-file-rotation defects in *tan1 air9* mutant plants, suggesting that defects that occur in mitosis influence the organization of non-dividing cells.

## INTRODUCTION

Correct division-plane orientation is key for patterning and growth across kingdoms. Because plant cells are confined by cell walls, division positioning is tightly regulated (Facette et al., 2019; Livanos and Müller, 2019; Rasmussen and Bellinger, 2018; Wu et al., 2018). Division-plane determination begins during S or G2, when the nucleus is repositioned within the cell (Facette et al., 2019; Frey et al., 2010; Wada, 2018; Yi and Goshima, 2020). Polarity is often established and maintained by nuclear repositioning and polar localization of proteins during asymmetric division (Facette et al., 2019; Guo et al., 2021; Kimata et al., 2016; Muroyama and Bergmann, 2019; Shao and Dong, 2016; Wada, 2018). Next, land-plant cells typically form a structure around the nucleus at the cell cortex called the preprophase band (PPB). The PPB is a ring of microtubules, microfilaments and associated proteins that marks the future position of the new cell wall, called the division site (Li et al., 2015; Pickett-Heaps et al., 1999; Rasmussen and Bellinger, 2018; Smertenko et al., 2017; Van Damme, 2009). Nuclear and PPB positioning often match division predictions based on cell geometry (Martinez et al., 2018; Moukhtar et al., 2019). PPB disassembly upon nuclear envelope breakdown precedes spindle formation (Dixit and Cyr, 2002). After chromosome separation, the phragmoplast forms from the anaphase spindle to direct new cell-wall synthesis. The phragmoplast is an antiparallel array of microtubules with plus ends facing the cell center (Ho et al., 2012; McMichael and Bednarek, 2013; Müller and Jürgens, 2016). Kinesins transport vesicles to form the cell plate (Lee and Liu, 2013; Smertenko et al., 2018). New microtubule nucleation expands the phragmoplast outwards until the cell plate contacts the division site (Gu and Rasmussen, 2022; Murata et al., 2013; van Oostende-Triplet et al., 2017).

Division-site-localized proteins, including TANGLED1 (TAN1), PHRAGMOPLAST ORIENTING KINESIN 1 (POK1), POK2, MICROTUBULE-ASSOCIATED PROTEIN 65-4 (MAP65-4), RAN GTPASE-ACTIVATING PROTEIN (RAN-GAP), MYOSIN VIII and KINESIN-LIKE CALMODULIN-BINDING PROTEIN (KCBP), remain at the cell cortex at the division site throughout cell division (Buschmann et al., 2015; Herrmann et al., 2018; Li et al., 2017; Lipka et al., 2014; Xu et al., 2008; Walker et al., 2007; Wu and Bezanilla, 2014). Many of these proteins are important for division-plane positioning, often during telophase. TAN1 is a division-site-localized protein required for phragmoplast guidance to the division site in maize (Martinez et al., 2017; Smith et al., 2001; Walker et al., 2007). TAN1 organizes microtubules at the cell cortex called cortical telophase microtubules, which are incorporated into the phragmoplast to direct its movement at the cell cortex (Bellinger et al., 2021). TAN1 binds and bundles microtubules *in vitro* (Martinez et al., 2020). Although the maize *tan1* mutant has misplaced divisions and stunted growth, *Arabidopsis thaliana tan1* mutants grow as well as wild-type plants and have minor division placement defects (Walker et al., 2007). Another division-site-localized protein, AIR9, also binds microtubules. AIR9 localizes to interphase cortical microtubule arrays, as well as colocalizing with the PPB and the phragmoplast, and localizing to the division site during late telophase (Buschmann et al., 2006). Similar to *tan1* single mutants, *air9* single mutants resemble wild-type plants (Buschmann et al., 2015). Due to their similar division-site localization, *tan1 air9* double mutants were generated in *Arabidopsis*. Combining mutations in both *tan1* and *air9* resulted in division-plane-positioning defects, stunted growth and root twisting in the differentiation zone (Mir et al., 2018). Although PPBs and phragmoplasts were both frequently misoriented in *tan1 air9* mutants, improper phragmoplast guidance was the primary defect (Mir et al., 2018). Transforming the *tan1 air9* double mutant with *TAN1–YFP* driven by the constitutive viral Cauliflower mosaic *CaMV35S* promoter rescued root growth, misoriented divisions and cell-file-rotation defects (Mir et al., 2018).

We hypothesized that TAN1 might also have a role in organizing interphase microtubules in elongating and differentiated cells, because *tan1 air9* mutants had aberrant cell-file rotation in the root-differentiation zone, minor defects in interphase microtubule organization and root growth defects that were enhanced by the microtubule-depolymerizing drug propyzamide (Mir et al., 2018). Cell-file-rotation phenotypes are often caused by mutations in microtubule-associated proteins or tubulin that alter the organization or stability of the interphase cortical microtubule array (Abe et al., 2004; Buschmann and Borchers, 2020; Buschmann et al., 2004; Hashimoto, 2015; Ishida et al., 2007; Nakajima et al., 2004; Sakai et al., 2008; Sedbrook et al., 2004; Shoji et al., 2004). For example, in several α-tubulin mutants, cell-file rotation occurred in hypocotyls and root-differentiation zones and in isolated cultured mutant cells (Abe et al., 2004; Buschmann et al., 2009; Ishida et al., 2007; Thitamadee et al., 2002). Cell-file twisting also occurs when cell elongation differs between epidermal and cortical cells. *Arabidopsis* treated with compounds that affect microtubule stability, such as oryzalin or propyzamide, have helical cell files due to cortical cell swelling and reduced longitudinal cell expansion (Furutani et al., 2000; Hashimoto, 2002). Therefore, defects in organ twisting are sometimes due to interphase microtubule disruption and likely independent of division-plane defects. However, several examples suggest that division-plane-orientation defects might lead to cell-file-rotation defects (Cnops et al., 2000; Wasteneys and Collings, 2009). Double mutants for two related receptor-like kinases have defects in division-plane orientation near the quiescent center and in the endodermis, and also have abnormal root skewing (Goff and Van Norman, 2021preprint). Therefore, it is possible that either mitotic or non-mitotic defects lead to aberrant growth and root-twisting defects.

To determine whether mitotic *TAN1* expression was sufficient to rescue root twisting in the differentiation zone of *tan1 air9* double mutants, we drove *TAN1* expression using the G2/M-phase-specific *KNOLLE* promoter (Lukowitz et al., 1996; Menges et al., 2005). KNOLLE is a syntaxin/Qa-SNARE required for cell-plate–vesicle fusion (Strompen et al., 2002; Völker et al., 2001). The *KNOLLE* promoter drove *TAN1* expression in mitotic cells, which rescued root growth and cell-file-rotation defects in the *tan1 air9* double mutant. Our results suggest that cell-file-rotation defects in the *tan1 air9* double mutant are likely due to defects that occur in actively dividing meristematic cells, and not due to a lack of TAN1 in non-dividing cells.

## RESULTS AND DISCUSSION

**Fig. 1.**
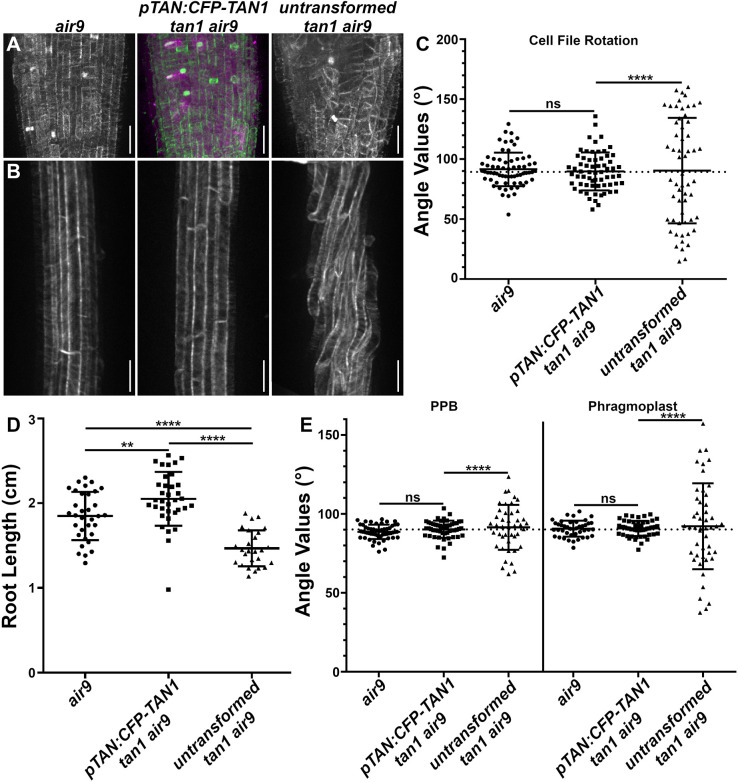
**TAN1 native promoter-driven TAN1 expression rescues the *tan1 air9* double mutant.** (A) Maximum-projection images of 20 1-µm *z*-stacks of root tips of *air9*, *pTAN:CFP–TAN1 tan1 air9* and untransformed *tan1 air9* plants expressing the microtubule marker *UBQ10:mScarlet–MAP4* (gray; green in the middle panel). *pTAN:CFP–TAN1* expression is shown in magenta (middle panel). Scale bars: 25 µm. (B) Maximum-projection images of 15 1-µm *z*-stacks of the differentiation zone of *air9*, *pTAN:CFP–TAN1 tan1 air9* and untransformed *tan1 air9* plants expressing *UBQ10:mScarlet–MAP4* (gray). Scale bars: 50 µm. (C) Cell-file-rotation angles of *air9*, *pTAN:CFP–TAN1 tan1 air9* and untransformed *tan1 air9* plants. *n*>11 plants for each genotype and *n*>57 cells for angle measurements. Cell-file-angle variances were compared with Levene's test due to the non-normal distribution. (D) Root-length measurements from 8 days after stratification of *air9*, *pTAN:CFP–TAN1 tan1 air9* and untransformed *tan1 air9* plants. *n*>25 plants for each genotype, compared by two-tailed unpaired *t*-test with Welch's corrections. (E) PPB and phragmoplast angle measurements in *air9*, *pTAN:CFP–TAN1 tan1 air9* and untransformed *tan1 air9* plants. *n*>9 plants for each genotype, *n*>41 cells for angle measurements. PPB and phragmoplast angle variations were compared with *F*-test. The mean±s.d. is indicated. ns, not significant; **P<0.01; ****P<0.0001.

We generated two independent TAN1 fluorescent protein fusions to determine whether *TAN1–YFP or CFP–TAN1* expressed by their native promoter would rescue the *tan1 air9* double mutant. Both constructs rescued the *tan1 air9* mutant. Previous studies have shown that *35S*-promoter-driven *TAN1* expression rescued *tan1 air9* mutants (Mir et al., 2018). We drove expression of *CFP–TAN1* and *TAN1–YFP* using the 1263 bp upstream of the start codon, i.e. *pTAN:CFP–TAN1* and *pTAN:TAN1–YFP*, respectively, and transformed or crossed them into the *tan1 air9* double mutant. Cell shape in the root tip (Fig. 1A) and cell-file rotation in the differentiation zone of *pTAN:CFP–TAN1 tan1 air9* plants were restored to those seen in the *air9* single mutant (Fig. 1B,C). Single *air9* mutants are indistinguishable from wild-type plants (Buschmann et al., 2015; Mir et al., 2018). Root-cell division primarily occurs at the root tip (the meristematic zone). Above that, non-dividing cells elongate in the elongation zone. Root hairs mark the differentiation zone, where root cells mature and differentiate into different cell types (Wachsman et al., 2015). *tan1 air9* mutant roots tend to twist left with variable transverse cell-wall angle values that skew above 90° (Mir et al., 2018). *pTAN:CFP–TAN1* rescued *tan1 air9* root growth, with *pTAN:CFP–TAN1*-expressing plants growing slightly longer than *air9* single mutants (Fig. 1D). *pTAN:TAN1–YFP* also fully rescued *tan1 air9* root growth and restored normal root-tip patterning (Fig. S1). Measuring PPB and phragmoplast angles is a metric for division-plane orientation, and PPB and phragmoplast angles were measured relative to the left-hand cell wall. *pTAN:CFP–TAN1* fully rescued PPB and phragmoplast-positioning defects in *tan1 air9* mutants, restoring angle variances close to 90° (Fig. 1E). This shows that mitotic expression of *TAN1* by its native promoter and a fluorescent protein fusion at either end of the TAN1 protein is sufficient for normal plant growth, including the expansion and patterning of non-dividing cells in the *tan1 air9* double mutant.

Previous fluorescence measurements of TAN1–YFP in wild-type lines expressing *pTAN:TAN1–YFP* demonstrated that the fluorescence signal above background levels was limited to the meristematic zone (Mir et al., 2018). We hypothesized that TAN1 accumulated at low but undetectable levels in interphase cells when driven by its native promoter. To test whether *TAN1* expression that was limited to mitotic cells influenced root growth and suppressed root twisting in the *tan1 air9* double mutant, we fused the *KNOLLE* promoter to *TAN1–YFP* (*pKN:TAN1–YFP*) and transformed it into the *tan1 air9* double mutant. The *KNOLLE* promoter is specifically expressed in G2/M and is contingent on the myeloblastosis (MYB) transcription factors MYB3R1 and MYB3R4, which promote mitosis-specific gene expression (Haga et al., 2011; Yang et al., 2021). Our prediction was that *pKN:TAN1–YFP* would fully rescue mitotic defects but not restore root growth or suppress aberrant cell-file rotation within the root-differentiation zone in the *tan1 air9* mutant.

**Fig. 2.**
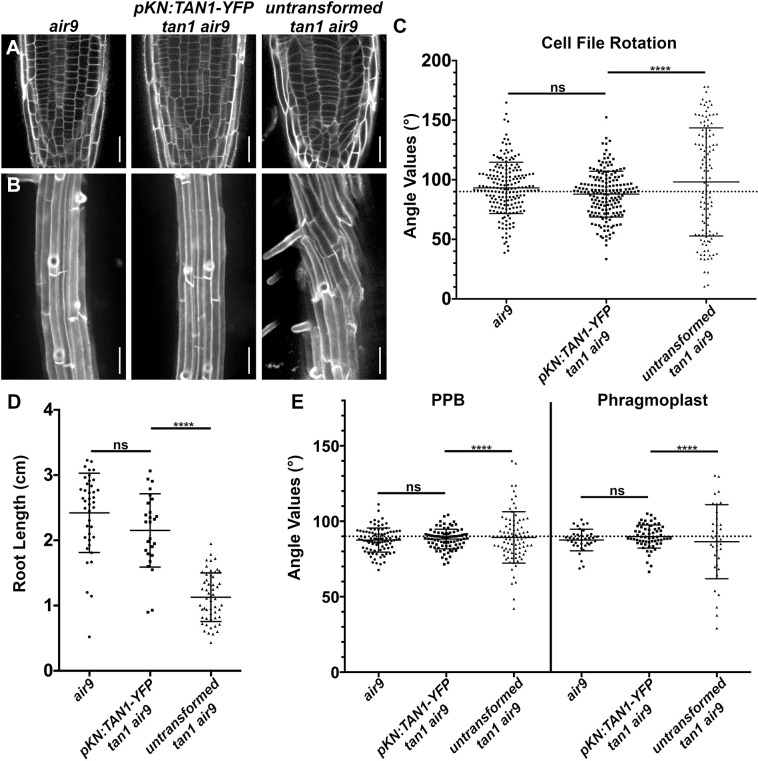
**Full rescue of the *tan1 air9* double mutant with G2/M-specific *KNOLLE* promoter-driven TAN1 expression*.*** (A) PI-stained cell walls in root tips of *air9*, *pKN:TAN1–YFP tan1 air9* and untransformed *tan1 air9* plants. Scale bars: 25 µm. (B) Maximum projection images of ten 1-µm *z*-stacks of PI-stained differentiation-zone root cell walls. Scale bars: 50 µm. (C) Cell-file-rotation angles of *air9*, *pKN:TAN1–YFP tan1 air9* and untransformed *tan1 air9* plants. *n*>23 plants for each genotype, *n*>114 cells for angle measurements. Cell-file-rotation angle variances were compared with Levene's test due to the non-normal distribution. (D) Root-length measurements from 8 days after stratification of *air9*, *pKN:TAN1–YFP tan1 air9* and untransformed *tan1 air9* plants. *n*>25 plants for each genotype, compared by two-tailed unpaired *t*-test with Welch's corrections. (E) PPB and phragmoplast angle measurements in *air9*, *pKN:TAN1–YFP tan1 air9* and untransformed *tan1 air9* plants. *n*>20 plants for each genotype, *n*>34 cells for angle measurements. PPB and phragmoplast angle variations were compared with *F*-test. The mean±s.d. is indicated. ns, not significant; ****P<0.0001.

**Fig. 3.**
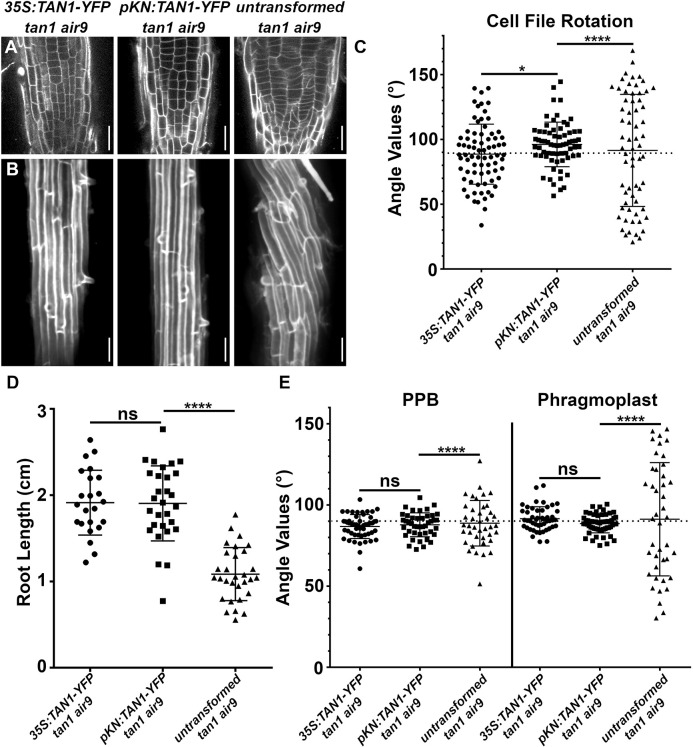
**Comparison between *KNOLLE* promoter-driven and *35S*-driven TAN1 expression-induced rescue of the *tan1 air9* double mutants.** (A) PI-stained root tips of *tan1 air9* mutants expressing *p35S:TAN1–YFP*, *pKN:TAN1–YFP* and untransformed plants. Scale bars: 25 µm. (B) Maximum projection images of ten 1-µm *z*-stacks of PI-stained differentiation-zone root cell walls of *tan1 air9* mutants expressing *p35S:TAN1–YFP* or *pKN:TAN1–YFP*, and untransformed *tan1 air9* mutants. Scale bars: 50 µm. (C) Cell-file-rotation angles of *tan1 air9* mutants expressing *p35S:TAN1–YFP* or *pKN:TAN1–YFP*, and untransformed *tan1 air9* mutants. *n*>13 plants for each genotype, *n*>64 cells for angle measurements. Angle variances were compared with Levene's test. (D) Root-length measurements from 8 days after stratification of *tan1 air9* mutants expressing *p35S:TAN1–YFP* or *pKN:TAN1–YFP*, and untransformed *tan1 air9* mutants. *n*>17 plants for each genotype, compared by two-tailed unpaired *t*-test with Welch's corrections. (E) PPB and phragmoplast angle measurements in *tan1 air9* double mutants expressing *p35S:TAN1–YFP* or *pKN:TAN1–YFP*, and untransformed *tan1 air9* mutants. *n*>12 plants for each genotype, *n*>39 cells for angle measurements. PPB and phragmoplast angle variations were compared with *F*-test. The mean±s.d. is indicated. ns, not significant; *P<0.05; ****P<0.0001.

**Fig. 4.**
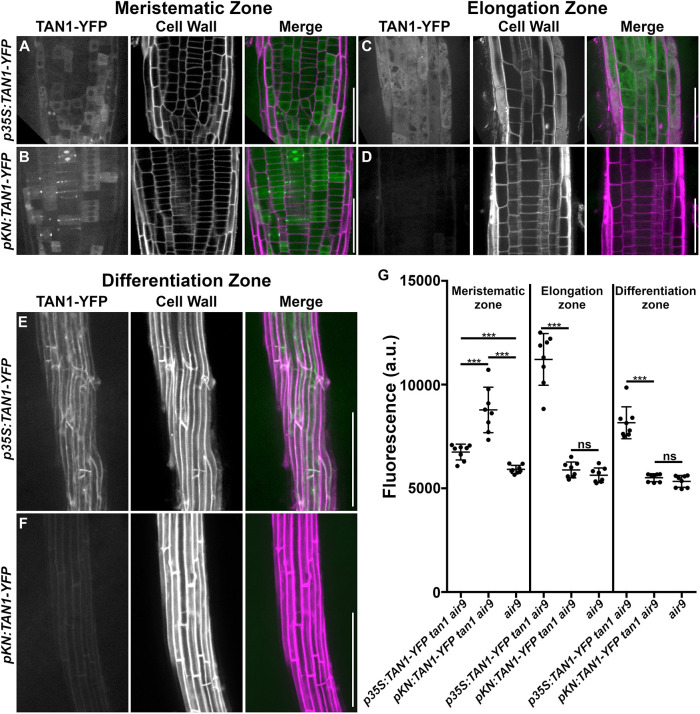
**Comparison of TAN1–YFP fluorescence intensity when its expression is driven by the constitutive *35S* promoter and the G2/M-specific *KNOLLE* promoter in *tan1 air9* roots.** (A–D) Micrographs of the meristematic zone (A,B), maximum-projection images of three 1-µm *z*-stacks of the elongation zone (C,D), and maximum-projection images of ten 1-µm *z*-stacks of the differentiation zone (E,F) of *tan1 air9* mutants expressing *p35S:TAN1–YFP* (A,C,E) or *pKN:TAN1–YFP* (B,D,F). Cell walls were stained with PI. Scale bars: 50 µm (root tip and elongation zones, A–D); 200 µm (differentiation zones, E,F). (G) TAN1–YFP fluorescence-intensity measurements (arbitrary units, a.u.) from the meristematic zone, elongation zone and differentiation zone of *tan1 air9* mutants expressing *p35S:TAN1–YFP* or *pKN:TAN1–YFP*, and *air9* mutants. *n*=8 plants for each genotype; values were compared with the Mann–Whitney *U*-test. The mean±s.d. is indicated. ns, not significant; ***P<0.001.

*pKN:TAN1–YFP* fully rescued the defects in *tan1 air9* mutants (Fig. 2; other independent lines shown in Fig. S2). This includes rescuing cell patterning and cell-file-rotation defects (Fig. 2A–C), root growth (Fig. 2D), and PPB and phragmoplast positioning (Fig. 2E). In addition, *pKN*-driven TAN1–YFP localized to the division site during mitotic stages, similar to *pTAN1*-driven CFP–TAN1 (Fig. S3). We compared phenotypes of *pKN:TAN1–YFP* to the *35S:TAN1–YFP* lines, which rescue the *tan1 air9* mutant (Mir et al., 2018). Both *35S:TAN1–YFP* and *pKN:TAN1–YFP* significantly rescued the *tan1 air9* double mutant (Fig. 3A,B). Root growth and PPB and phragmoplast angles were equivalent in *tan1 air9* plants expressing *pKN:TAN1–YFP* or *35S:TAN1–YFP* (Fig. 3D,E). However, *pKN:TAN1–YFP* reduced cell-file-rotation variability slightly more than *35S:TAN1–YFP* (Fig. 3C). This suggests that expressing *TAN1* in dividing cells is sufficient to fully rescue the *tan1 air9* double mutant. To determine why rescue with the *KNOLLE* promoter resulted in less cell-file-rotation variance, we measured TAN1–YFP fluorescence intensities in the *35S:TAN1–YFP* (Fig. 4A,C,E) and *pKN:TAN1–YFP* lines (Fig. 4B,D,F). *pKN:TAN1–YFP* was expressed strongly in the meristematic zone of root tips (Fig. 4B; Fig. S4), often showing TAN1–YFP fluorescence in recently divided cells, similar to native-promoter-driven accumulation (Fig. 1; Figs S1 and S4; Mir et al., 2018). Indeed, TAN1–YFP accumulated at higher levels in the meristematic zone when expression was driven by the *KNOLLE* promoter (Fig. 4G; Fig. S4). However, unlike TAN1–YFP from *p35S:TAN1–YFP* (Fig. 4E,F), TAN1–YFP did not accumulate above background levels in the elongation and differentiation zone of roots expressing *pKN:TAN1–YFP* (Fig. 4F,G; Fig. S4). Lack of TAN1–YFP outside the meristematic zone and more complete rescue of *tan1 air9* cell-file rotation by *pKN:TAN1–YFP* suggests that TAN1 is not required in elongating and differentiating cells. In other words, cell-file-rotation defects might be a consequence of defects that occur within the root meristematic zone either during mitosis or shortly afterwards.

Another example of defects in mitotic expression and division-plane positioning affecting non-dividing cell organization occurs in the MYB-activated GRAS [GIBBERELLIC ACID INSENSITIVE (GAI), REPRESSOR OF GAI and SCARECROW]-type transcription factor *scarecrow-like 28-3* (*scl28-3)* mutant. G2/M-specific gene expression controlled by SCL28 is important for mitotic progression and division-plane positioning. *scl28-3* mutants have both misoriented divisions and root twisting (Goldy et al., 2021).

How do defects that occur within the meristematic zone influence the patterning or shape of non-dividing, differentiating root cells and root growth? Our hypothesis is that misshapen cells and improper division-plane orientation in *tan1 air9* double mutants cause the uneven distribution of mechanical stresses across the root, which then triggers cell-wall-integrity responses that limit growth and alter root organization. Cell-wall stress patterns depend on cell geometry and the mechanical properties of cell walls (Cosgrove, 2018; Hamant and Haswell, 2017; Whitewoods and Coen, 2017; Schopfer, 2006).

Division-plane positioning is a way in which plants can respond to mechanical stress (Chakrabortty et al., 2018; Louveaux et al., 2016). Cell division relieves mechanical stress by creating smaller cells with less surface area; furthermore, divisions along maximal tensile stress promote growth homogeneity (Alim et al., 2012; Sapala et al., 2018). Microtubules often align parallel to maximal tensile stress (Hamant et al., 2008; Heisler et al., 2010; Sampathkumar et al., 2014; Uyttewaal et al., 2012) and cortical-microtubule alignment often influences PPB placement (Louveaux et al., 2016; Rasmussen et al., 2013; Wick and Duniec, 1983). However, division-plane positioning is disrupted in mutants with division-plane-orientation defects. Although *tan1 air9* cells may perceive mechanical stress, phragmoplast guidance defects prevent the construction of new cell walls in an orientation that minimizes mechanical stress. Abnormal stresses are perceived by receptor-like kinases involved in the cell-wall-integrity response. Cell-wall-integrity responses trigger slow growth, upregulation of stress responses and changes in cell morphogenesis, (Buschmann and Borchers, 2020; Caño-Delgado et al., 2003; Gonneau et al., 2018; Hématy et al., 2007; Wolf et al., 2014), which might contribute to the stunted growth and twisted cell files observed in the *tan1 air9* double mutant.

## MATERIALS AND METHODS

### Plasmid construction

*pKN:TAN1–YFP* was generated by amplifying 2152 bp of the 5′ end of the *KNOLLE* (AT1G08560) promoter from Columbia with the primers pKN-5′SacI Fw and pKN-5′EcoRI Rw (see Table S1 for a list of primers). EcoRI and StuI double digestion was used to introduce the *KNOLLE* promoter into pEZT-NL (a gift from David Ehrhardt, Carnegie Institute) containing the TAN1 coding sequence (CDS). The primers 35SpKN5′ Fw and YFP XhoI Rw were used to amplify *pKN:TAN1–YFP*, then XhoI and StuI double digestion was used to clone *pKN:TAN1–YFP* into pEGAD, a gift from Prof. Sean Cutler (University of California, Riverside).

*pTAN:CFP–TAN1* was created by overlapping PCR. The 1263 bp 5′ sequence upstream of genomic *TAN1* was amplified using *Np:AtTAN–YFP* (Walker et al., 2007) as a template with the primers NpTANSacIFor and NpTANceruleanRev. Cerulean fluorescent protein (CFP) was amplified using the Cerulean CDS in pDONR221P4r/P3r, a kind gift from Prof. Anne Sylvester (University of Wyoming), as a template with the primers NpTANceruleanFor and CeruleanpEarleyRev. The TAN1 CDS was amplified using *35S:YFP–TAN1* in pEarley104 (*Arabidopsis* Biological Resource Center; Earley et al., 2006) as a template with the primers CeruleanpEarleyFor and pEarleyOCSPstIRev. The 1263 bp TAN1 native promoter and the CFP and TAN1 CDSs were combined to create *pTAN:CFP–TAN1* by overlapping PCR with the primers NpTANSacI and pEarleyOCSPstIRev. SacI and PstI double digestion was used to subclone *pTAN:CFP–TAN1* into pJHA212G, a kind gift from Prof. Meng Chen (University of California, Riverside).

### Generation of transgenic lines

Transgenic *Arabidopsis* lines were generated using *Agrobacterium tumefaciens*-mediated floral dip transformation as described previously (Clough and Bent, 1999). Previously described *tan1 air9* mutants (Mir et al., 2018), *csh-tan* (*TAN1*, AT3G05330; Walker et al., 2007) and *air9-31* (*AIR9*, AT2G34680; Buschmann et al., 2015), were used for floral dip transformation of *pKN:TAN1–YFP* and T1 transgenic plants were subsequently selected on half-strength Murashige and Skoog (1/2 MS) medium (MP Biomedicals; Murashige and Skoog, 1962) plates containing 15 μg/ml glufosinate (Finale; Bayer). TAN1–YFP signal in T1 plants was confirmed by confocal microscopy before being transferred to soil and selfed. The genotypes of *csh-tan1 air9-31* transformants was confirmed using the primers AIR9_cDNA 2230 F and AIR9 gnm7511 R (to identify *AIR9* wild type); AIR9 gnm7511 R and Ds5-4 (to identify T-DNA insertion in *AIR9*); ATLP and AtTAN 733-CDS Rw (to identify TAN1 wild type); and AtTAN 733-CDS Rw and Ds5-4 (to identify T-DNA insertion in *TAN1*). The microtubule marker *CFP–TUBULIN* (Kirik et al., 2007), a kind gift from David Ehrhardt (Carnegie Institute) was crossed into *pKN:TAN1–YFP tan1 air9* plants using *tan1 air9 CFP–TUBULIN* plants (Mir et al., 2018).

*air9-5 tan-mad* Columbia/Wassilewskija double mutants (Mir et al., 2018) expressing the microtubule marker *UBQ10:mScarlet–MAP4* (Pan et al., 2020), a kind gift from Prof. Zhenbiao Yang (University of California, Riverside), was used for floral dip transformation of *pTAN1:CFP–TAN1* and selected on 1/2 MS plates containing 100 μg/ml gentamicin (Thermo Fisher Scientific). T1 seedlings were screened for mScarlet and CFP signals and then transferred to soil to self.

### Growth conditions and root length measurements

Plates containing 1/2 MS containing 0.5 g/l MES buffer (Thermo Fisher Scientific), pH 5.7, and solidified with 0.8% agar (Thermo Fisher Scientific) were used to grow *Arabidopsis* seedlings. *tan1 air9* transgenic lines expressing *p35S:TAN1–YFP* (T3), *pKN:TAN1–YFP* (T2) and *pTAN:CFP–TAN1* (T2) were used for root length experiments. At least three biological replicates were used for each root growth assay. Five to seven 1/2 MS plates were used for each replicate. Twelve to 15 seeds were sown in a single level line on each plate, with untransformed *tan1 air9* double mutants and *air9* single mutants sown on plates alongside double mutants expressing *TAN1* constructs. Seeds were stratified on plates in the dark at ∼4°C for 2–5 days. After stratifying, plates were positioned vertically in a growth chamber (Percival) with a 16-h/8-h light/dark cycle and temperature set to 22°C. Each biological replicate was placed in the growth chamber on different days. Eight days after stratification, plates were scanned (Epson) and root lengths were measured using FIJI (ImageJ, http://fiji.sc/). Transgenic seedlings were screened for fluorescence by confocal microscopy to identify seedlings expressing YFP, CFP and mScarlet translational fusions. Each root growth experiment had a minimum of three biological replicates. Statistical analysis of root length was performed using two-tailed unpaired Welch's *t*-test with Prism (GraphPad) and replicates were checked for discrepancies in statistical significance before pooling replicates for analysis. Root length plots were created using Prism (GraphPad).

To assess the ability of *TAN1* driven by its native promoter to rescue the *tan1 air9* double mutant, *Np:AtTAN–YFP* (Walker et al., 2007) was crossed to *tan-mad air9-5* double mutants. The progeny of *pTAN1:TAN1–YFP tan-mad*/+ *air9-5*/+ plants were sown on 1/2 MS media and grown as described above. The seedlings were screened by confocal microscopy for the presence of TAN1–YFP and then collected for genotyping. Seedlings were genotyped with primers AtExon1_1For and At255AfterStopRev (to identify wild-type *TAN1*); JL202 and ATLP (to identify T-DNA insertion in *TAN1*); AIR9-5RP and AIR9-5LP (to identify wild-type *AIR9*); and LBb1.3 and AIR9RP (to identify T-DNA insertion in *AIR9*) (Table S1). The root lengths of *tan1 air9* double mutants expressing *pTAN1:TAN1–YFP* were compared to those of *tan1 air9* double mutants and *air9* single mutant siblings lacking *pTAN1:TAN1–YFP*. *air9* single mutants used for root length analysis included *air9/air9 TAN1/TAN1* and *air9/air9 TAN1/tan1* plants.

### Confocal microscopy

Imaging and screening were performed using Micromanager software (https://micro-manager.org/) running on an inverted Ti Eclipse microscope (Nikon) with a motorized stage (ASI Piezo) and a spinning-disk confocal microscope (Yokogawa W1) built by Solamere Technology. Solid-state lasers (Obis) were used with standard emission filters (Chroma Technology). Excitation at 445 nm, emission at 480/40 nm (for CFP translational fusions); excitation at 514 nm, emission at 540/30 nm (for YFP translational fusions); and excitation at 561 nm, emission at 620/60 nm [for propidium iodide (PI) and mScarlet–MAP4] were used. The 20× objective with 0.75 numerical aperture (NA) is an air objective. The 60× objective was used with perfluorocarbon immersion liquid (RIAAA-6788, Cargille) and has 1.2 NA.

### Measurements of PPB and phragmoplast angles and cell-file rotation

All angle data was gathered from at least three biological replicates. Each replicate consisted of five to seven 1/2 MS plates with 12–15 seeds sown on each plate. Four to five seeds of each genotype were sown on each plate to ensure growing conditions were identical. Each replicate was transferred from stratifying to the growth chamber on independent days. Seedlings were imaged at 8 days after stratification. The 20× objective was used to collect images of the differentiation zone for cell-file angles and the 60× objective to collect images of root tips expressing a microtubule marker (*CFP–TUBULIN* or *mScarlet–MAP4*) for PPB and phragmoplast angles. The differentiation zone was identified by the presence of root hairs. Angles were measured using FIJI. Cell-file angles were measured from the left-hand side of the cell, taking the angle between the long axis of the root and the transverse cell wall in the differentiation zone. PPB and phragmoplast angles were measured as the angle between the left-hand cell wall and the PPB or phragmoplast. *CFP–TUBULIN*-expressing seedlings were stained with 10 μM PI to stain cell walls for 1 min before destaining in distilled water prior to imaging. Each angle measurement represents a single angle measured from one cell.

Statistical analyses were performed using Excel (Microsoft Office) and Prism (GraphPad). To compare normally distributed variance of PPB and phragmoplast angles, *F*-test was used. Levene's test was used to compare variances of cell-file angle measurements because *tan1 air9* cell-file angles are non-normally distributed due to left-hand twisting of the roots. Angle variance across biological replicates was checked before pooling data.

### Fluorescence intensity measurements

*air9*, *35S:TAN1–YFP tan1 air9* and *pKN:TAN1–YFP tan1 air9* plants were grown on 1/2 MS plates as described above. Eight days after stratification, plants were imaged by confocal microscopy using identical settings. Root tips were imaged using the 60× objective. The median fluorescence intensity of a 116,001.5 µm^2^ area was measured from multiple individual plants of each genotype. Each fluorescence measurement represents the median fluorescence from a single meristematic zone from one plant. Elongation zone and differentiation zone images were taken with the 20× objective and the median fluorescence intensity of a 12,323.4 µm^2^ area was measured from multiple individual plants of each genotype. Each fluorescence measurement represents the median fluorescence from a single elongation or differentiation zone from one plant. For Fig. S4, the same imaging conditions and 20× objective for five 8-day-old seedlings of each genotype were used to collect root images, which were stitched together in FIJI for *air9* single mutant and *tan1 air9* double mutants expressing *pTAN:TAN1–YFP*, *pKN:TAN1–YFP*, *35S:YFP–TAN1* and *35S:TAN1–YFP*.

## Supplementary Material

10.1242/joces.260127_sup1 Supplementary information
