# Supplementary material for: Defects in division plane positioning in the root meristematic zone affect cell organization in the differentiation zone
Source: J Cell Sci. 2022 Sep 29;135(19):jcs260127. doi: 10.1242/jcs.260127 (PMC9658997; doi:10.1242/jcs.260127)
Supplement: Supplementary information [file joces-135-260127-s1.pdf]

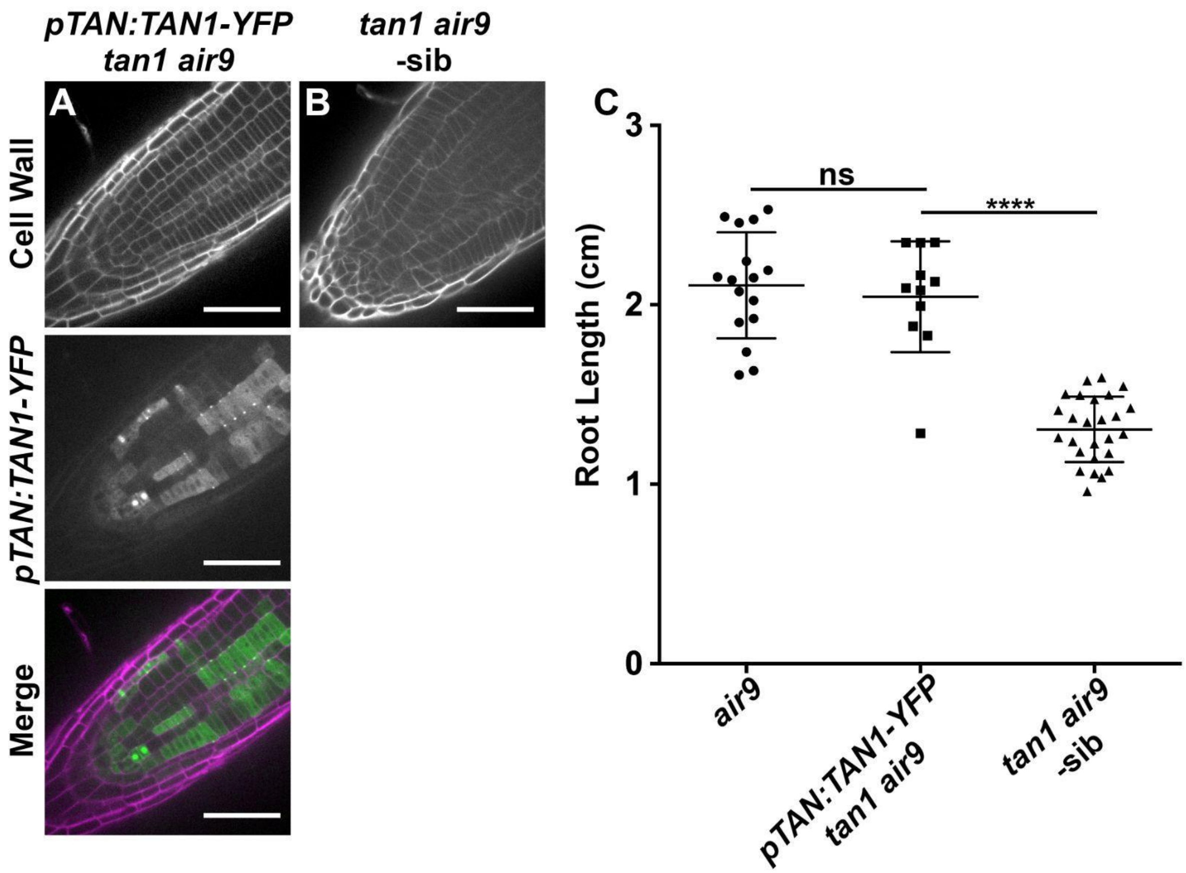

Fig. S1. *TAN1-YFP* expressed by its native promoter (*pTAN:TAN1-YFP*) rescues *tan1 air9* double mutant root growth. Confocal images of propidium iodide-stained roots of *tan1 air9* plants. A) A *tan1 air9* plant expressing *pTAN:TAN1-YFP*. B) A negative sibling *tan1 air9* plant. Bars = 50 µm. C) Root length measurements from 8 days after stratification of *air9* single mutants (left), *pTAN1:TAN1-YFP tan1 air9* double mutants (middle), and *tan1 air9* double mutants (right). n > 10 plants for each genotype, compared by two-tailed t-test with Welch's correction. ns indicates not significant, **** P-value <0.0001. Mean ± s.d. indicated.

Journal of Cell Science • Supplementary information

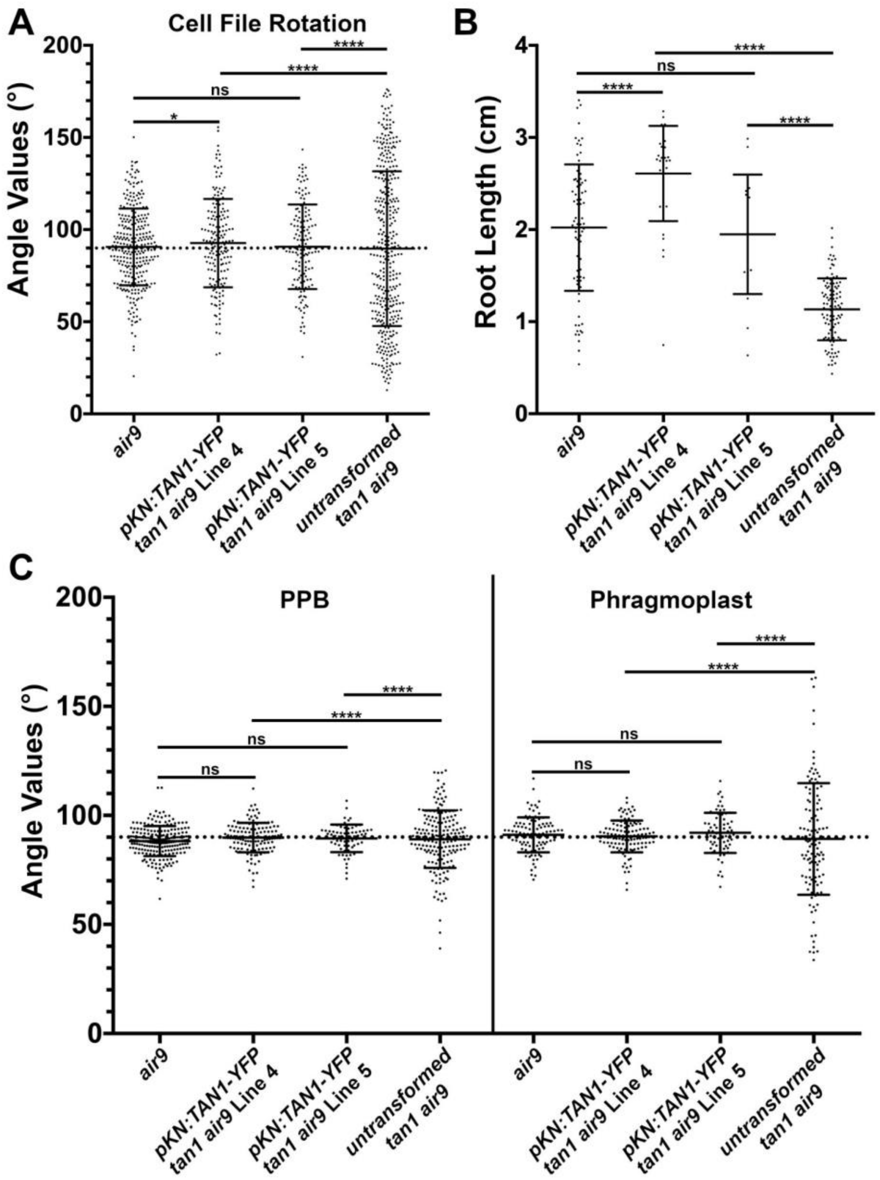

Fig. S2. *pKN:TAN1-YFP tan1 air9* lines show significant rescue compared to untransformed *tan1 air9*. A) Cell file rotation angles of *air9* single mutants (left), two transgenic lines expressing pKN*:TAN1-YFP* in the *tan1 air9* double mutant designated as line 4 (center left) and line 5 (center right) and untransformed plants (right), n >17 plants for each genotype. N > 146 cells for angle measurements. Angle variances were compared with Levene's test. B) Root length measurements from 8 days after stratification of *air9* single mutants (left), two transgenic lines expressing pKN*:TAN1-YFP* in the *tan1 air9* double mutant (middle), and untransformed plants (right), n > 21 plants for each genotype, compared by two-tailed t-test with Welch's correction. C) PPB and phragmoplast angle measurements in dividing root cells of *air9* single mutants (left), two transgenic lines expressing pKN*:TAN1-YFP* in the *tan1 air9* double mutant (middle), and untransformed plants (right), n > 15 plants for each genotype. N > 69 cells for angle measurements. Angle variations compared with F-test. ns indicates not significant, * P-value < 0.05, **** P-value <0.0001. Mean ± s.d. indicated.

Journal of Cell Science • Supplementary information

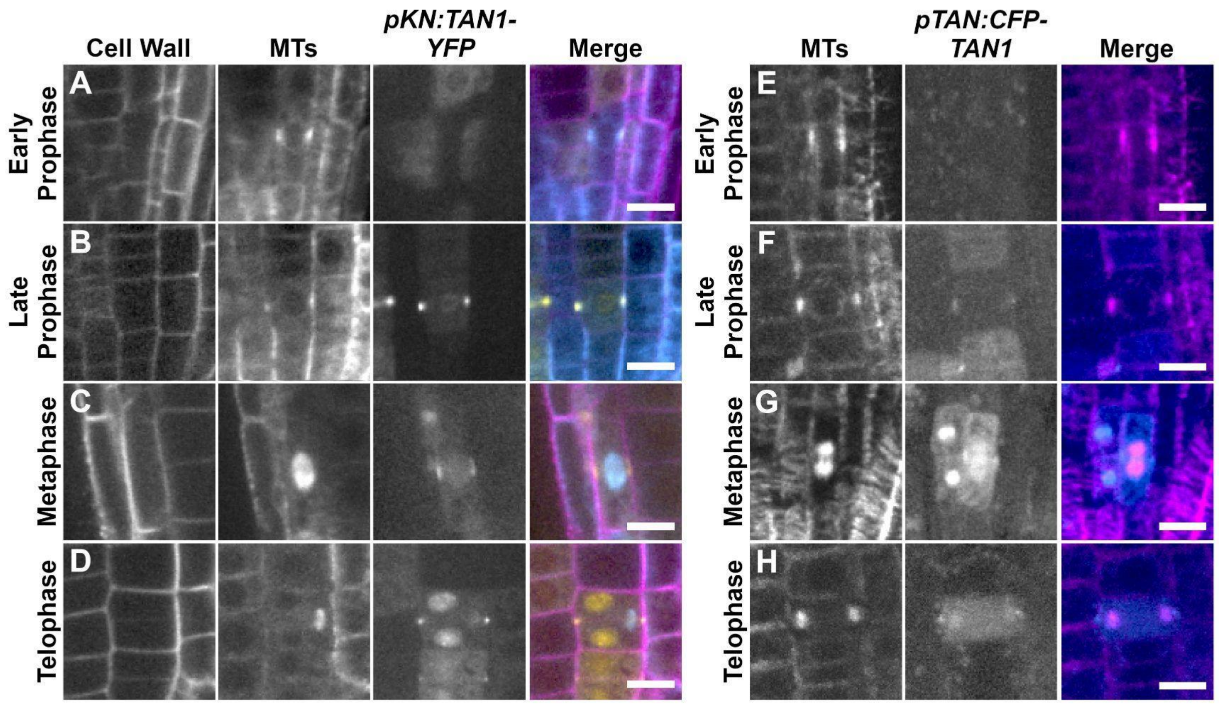

Fig. S3. Division site localization of TAN1-YFP driven by the *KNOLLE* promoter (*pKN:TAN1-YFP*) and CFP-TAN1 driven by the *TAN1* promoter (*pTAN:CFP-TAN1*) in *tan1 air9* double mutants. A-D) Confocal images of propidium iodide-stained (Cell Wall) roots of *tan1 air9* plants expressing *pKN:TAN1-YFP* and *CFP-TUBULIN* (MTs) in dividing root tip cells. E-F) Maximum projections of 3 1-µm Z-stacks of *tan1 air9* plants expressing *pTAN:CFP-TAN1* and the microtubule (MTs) marker *UBQ10:mScarlet-MAP4* in dividing root tip cells. Representative images of cells with (A&E) broad early PPBs, (B&F) late narrow PPBs, (C&G) metaphase spindles, and (D&H) phragmoplasts. Bars = 10 µm.

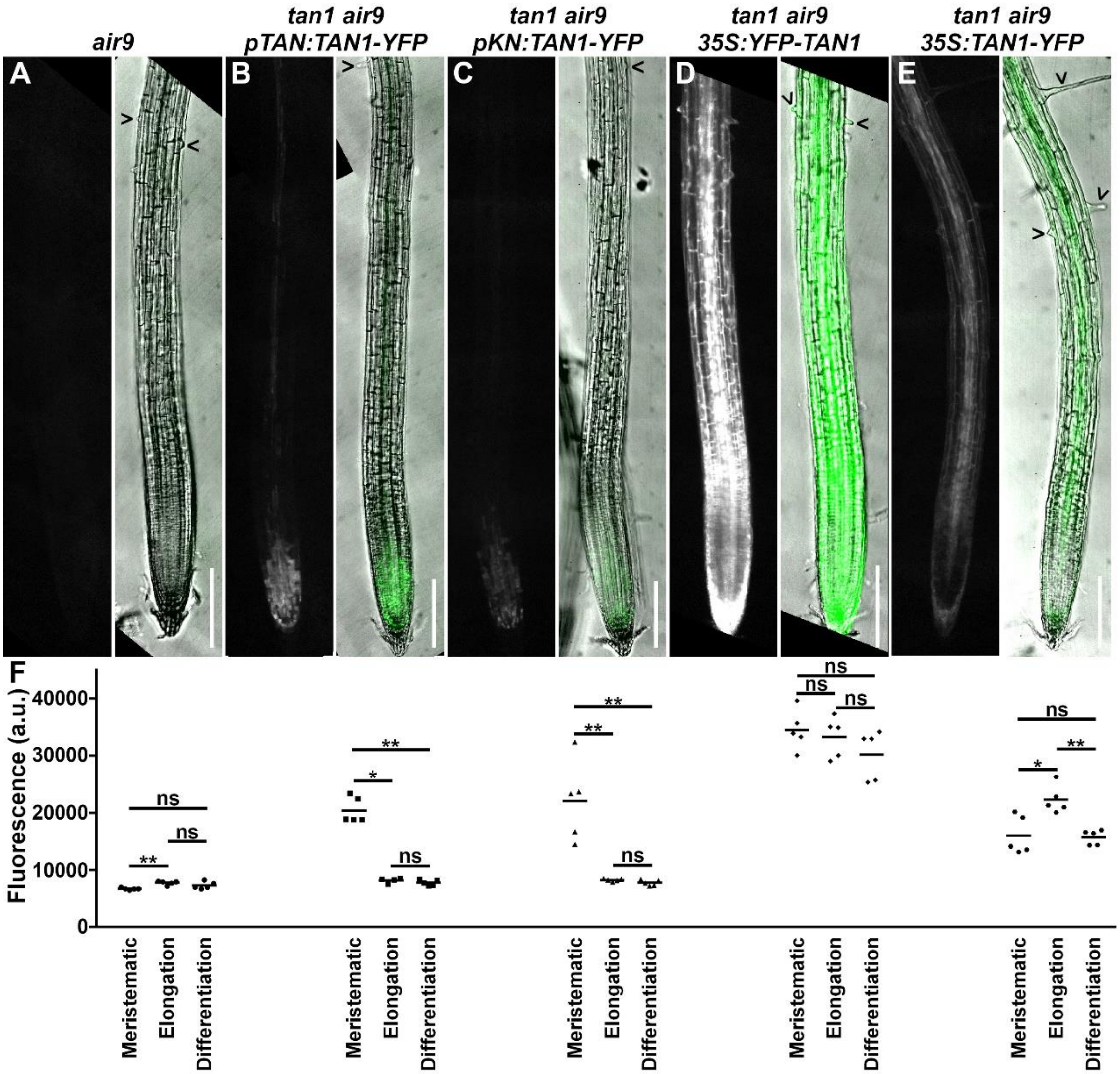

Fig. S4. YFP fluorescence in the roots of (A) an *air9* single mutant and *tan1 air9* double mutants expressing (B) *pTAN:TAN1-YFP*, (C) *pKN:TAN1-YFP*, (D) *35S:YFP-TAN1*, and (E) *35S:TAN1-YFP*. A-E) Left panels show YFP channel only and right panels show YFP signal in green overlaid on bright-field root images. Black arrows indicate root hairs. Bars = 200 µm. F) YFP fluorescence-intensity measurements (arbitrary units, a.u.) from the meristematic zone, elongation zone, and differentiation zone of *air9* single mutants and *tan1 air9* mutants expressing *pTAN:TAN1-YFP*, *pKN:TAN1-YFP*, *p35S:YFP-TAN1*, and *p35S:TAN1-YFP*. n=5 plants for each genotype, fluorescence compared with Mann-Whitney U test. * P-value <0.05,** P-value <0.01, and ns indicates not significant. Fluorescence in the elongation and differentiation zones of *tan1 air9* plants expressing *pTAN:TAN1-YFP* (P-value > 0.1) and *pKN:TAN1-YFP* (P-value > 0.05) was not significantly different from *air9* single mutants. Fluorescence in the root tips of *pTAN:TAN1-YFP*, *pKN:TAN1-YFP*, *35S:YFP-TAN1*, and *35S:TAN1-YFP* expressing plants was significantly different compared to *air9* root tips (P-value = 0.008). Mean ± s.d indicated.

Journal of Cell Science • Supplementary information

Table S1. Primers used for cloning and genotyping

Primer Name	Sequence
ATRP	ATCTCTTAGGAACCAAAACCGGACGCTGT
ATLP	GATCCGTTACGAAAGTGAACACCTTTATC
JL202	CATTTTATAATAACGCTGCGGACATCTAC
AIR9-5RP	TGGATCAGCTGCAACATTATTC
AIR9-5LP	ATTAACATTTTGCAACGCAGG
LBb1.3	ATTTTGCCGATTTCGGAAC
Ds5-4	TACGATAACGGTCGGTACGG
AtTAN 733-CDS Rw	AAATAGAGGGTTCGGAAAAAGAACC
AIR9 gnm7511 R	CCTCCAGTATATGAAGCAACAAAGC
AIR9_cDNA 2230 F	GATGAGGAATATATGTTATCTTTAGATG
pKN-5’SacI Fw	GAGGAGCTCCAGAAGAAAAAGAAAAAGTTCTC
pKN-5’EcoRI Rw	TAAGCGGAATTCCTTTTTCACCTGAAA
35SpKN5’ Fw	ACCCACAGATGGTTAGAGagg
YFP XhoI Rw	ATAATGCTCGAGAGAGTCGCG
NpTANSacIFor	GTATGAGCTCCGGTAGAGTTGAACCAG
NpTANceruleanRev	CCTCGCCCTTGCTCACCATCTTCTATATATATTTTCTTTA
NpTANceruleanFor	TAAAGAAAATATATATAGAAGATGGTGAGCAAGGGCGAGG
CeruleanpEarleyRev	GGCCCGCGGTACCGTCCTTGTACAGCTCGTCCATGC
CeruleanpEarleyFor	GCATGGACGAGCTGTACAAGGACGGTACCGCGGGCC
pEarleyOCSPstIRev	CCATCTGCAGCTGCTGAGCCTCGACAT
AtExon1_1For	CTCAACTCAGATCTTCTCAAGGAAACG
At255AfterStopRev	GCATAGTGGTACCCTCAAATTACACC

Journal of Cell Science • Supplementary information
